# First‐Line Alectinib Versus Ceritinib With or Without Brain Radiotherapy in ALK‐Positive Non‐Small Cell Lung Cancer with Brain Metastases: A Real‐World Multicenter Study from Vietnam

**DOI:** 10.1111/1759-7714.70325

**Published:** 2026-06-18

**Authors:** Hoang Gia Nguyen, Xuan Hau Nguyen, Phuong Nguyen, Thu Huong Nguyen, Quang Bui Vinh

**Affiliations:** ^1^ Lung and Breast Medical Oncology Department Hanoi Oncology Hospital Hanoi Vietnam; ^2^ Department of Oncology Hanoi Medical University Hanoi Vietnam; ^3^ Department of Medical Oncology Vietnam National Cancer Hospital – K Hospital Hanoi Vietnam; ^4^ Department of Radiation Oncology Hanoi Oncology Hospital Hanoi Vietnam

**Keywords:** alectinib, anaplastic lymphoma kinase, brain metastases, carcinoma, ceritinib, non‐small‐cell lung

## Abstract

**Background:**

This study compared the efficacy of first‐line alectinib versus ceritinib and evaluated the role of upfront brain radiotherapy (RT) in patients with ALK‐positive non‐small cell lung cancer (NSCLC) and brain metastases (BM).

**Methods:**

We retrospectively analyzed 108 patients with ALK‐positive NSCLC and baseline BM treated with first‐line alectinib (*n* = 42) or ceritinib (*n* = 66) at two Vietnamese centers between 2019 and 2025. Intracranial and systemic responses were assessed by RANO‐BM and RECIST v1.1 criteria, respectively. Survival outcomes were analyzed using the Kaplan–Meier method and multivariable Cox regression.

**Results:**

First‐line alectinib demonstrated superior intracranial disease control compared with ceritinib (iDCR 97.6% vs. 83.3%, *p* = 0.026; median iPFS 40.4 vs. 17.2 months, univariable HR 0.46, 95% CI 0.26–0.82, *p* = 0.005). Systemic PFS was also significantly longer with alectinib (35.9 vs. 11.5 months, univariable HR 0.38, 95% CI 0.22–0.65, *p* = 0.001). Exploratory subgroup analyses suggested differential effects of upfront brain RT by TKI, but interaction tests were non‐significant (*p* = 0.642 for iPFS, *p* = 0.837 for systemic PFS). In multivariable analysis, alectinib remained an independent predictor of better iPFS (HR 0.38, 95% CI 0.20–0.70, *p* = 0.002) and systemic PFS (HR 0.34, 95% CI 0.20–0.59, *p* = 0.001). Grade ≥ 3 adverse events occurred in 19.0% of patients with alectinib versus 34.8% with ceritinib (*p* = 0.068).

**Conclusions:**

In this real‐world Vietnamese cohort, first‐line alectinib showed superior intracranial and systemic efficacy over ceritinib. In resource‐limited settings, ceritinib combined with upfront brain RT is a reasonable clinical alternative. The differential benefit of brain RT between the two TKIs remains hypothesis‐generating.

## Graphical abstract

Alectinib demonstrated superior intracranial disease control and systemic efficacy compared with ceritinib in Vietnamese patients with ALK‐positive NSCLC and brain metastases. Upfront brain radiotherapy appeared to provide greater benefit when combined with ceritinib than with alectinib (exploratory, interaction *p* > 0.6). Alectinib is preferred when available; ceritinib plus upfront brain RT is a reasonable alternative in resource‐limited settings.

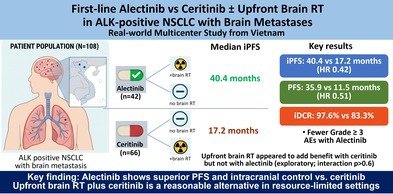

## 1 Introduction

Anaplastic lymphoma kinase (ALK) rearrangements occur in approximately 3%–7% of patients with non‐small cell lung cancer (NSCLC) and are more common in younger individuals, never/light smokers, and patients with adenocarcinoma histology [1]. Brain metastases are present at diagnosis in 26%–42% of ALK‐positive cases, as demonstrated in pivotal randomized clinical trials [2, 3, 4, 5] and frequently develop during the disease course [6], substantially worsen prognosis and quality of life [7].

ALK tyrosine kinase inhibitors (ALK‐TKIs) have become the standard of care for advanced ALK‐positive NSCLC [8, 9]. Second‐generation ALK‐TKIs, such as alectinib and ceritinib, provide superior blood–brain barrier penetration and greater outcomes than first‐generation agents [10]. However, access to these novel drugs remains limited in many developing countries, including Vietnam, primarily due to their high cost and lack of reimbursement under the national health insurance scheme. Consequently, real‐world data on the performance of these two agents in Vietnamese patients are currently scarce. Moreover, Southeast Asian populations are substantially underrepresented in the ALK‐positive NSCLC literature, which is dominated by cohorts from North American, European, and East Asian countries. Real‐world evidence from Vietnam and other middle‐income countries is essential to validate the generalizability of trial findings and inform treatment algorithms in resource‐constrained healthcare settings.

Furthermore, local treatments such as radiotherapy continue to play an important role in the management of brain metastases, even in the era of systemic therapies [11]. Radiotherapy can be synergistic with TKIs by improving local control, reducing BBB constraints, and addressing oligoprogressive sites where resistant clones emerge [12]. However, these local therapies carry risks of treatment‐related complications, including neurocognitive decline and radiation‐induced leukoencephalopathy [13, 14]. The role of combining brain RT with second‐generation ALK‐TKIs remains unclear, given the limited enrollment of patients with baseline brain metastases in pivotal trials and the absence of dedicated subgroup analyses. In routine clinical practice, upfront brain RT is frequently administered prior to the initiation of TKI therapy. Consequently, the optimal role of upfront cranial radiation when combined with second‐generation ALK‐TKIs remains an area of ongoing investigation and clinical uncertainty.

This multicenter retrospective cohort study evaluated the real‐world effectiveness of first‐line alectinib versus ceritinib in Vietnamese patients with ALK‐positive NSCLC and baseline brain metastases. In addition, we aimed to compare clinical outcomes between patients receiving TKI monotherapy and those receiving TKI combined with upfront cranial irradiation.

## 2 Materials and Methods

### 2.1 Study Design and Patients

This retrospective cohort study was conducted at two tertiary oncology hospitals in Vietnam: Vietnam National Cancer Hospital and Hanoi Oncology Hospital. Patients were identified from electronic medical records between January 1, 2019 and December 31, 2025. The study followed the Strengthening the Reporting of Observational Studies in Epidemiology (STROBE) guidelines.

**FIGURE 1:**
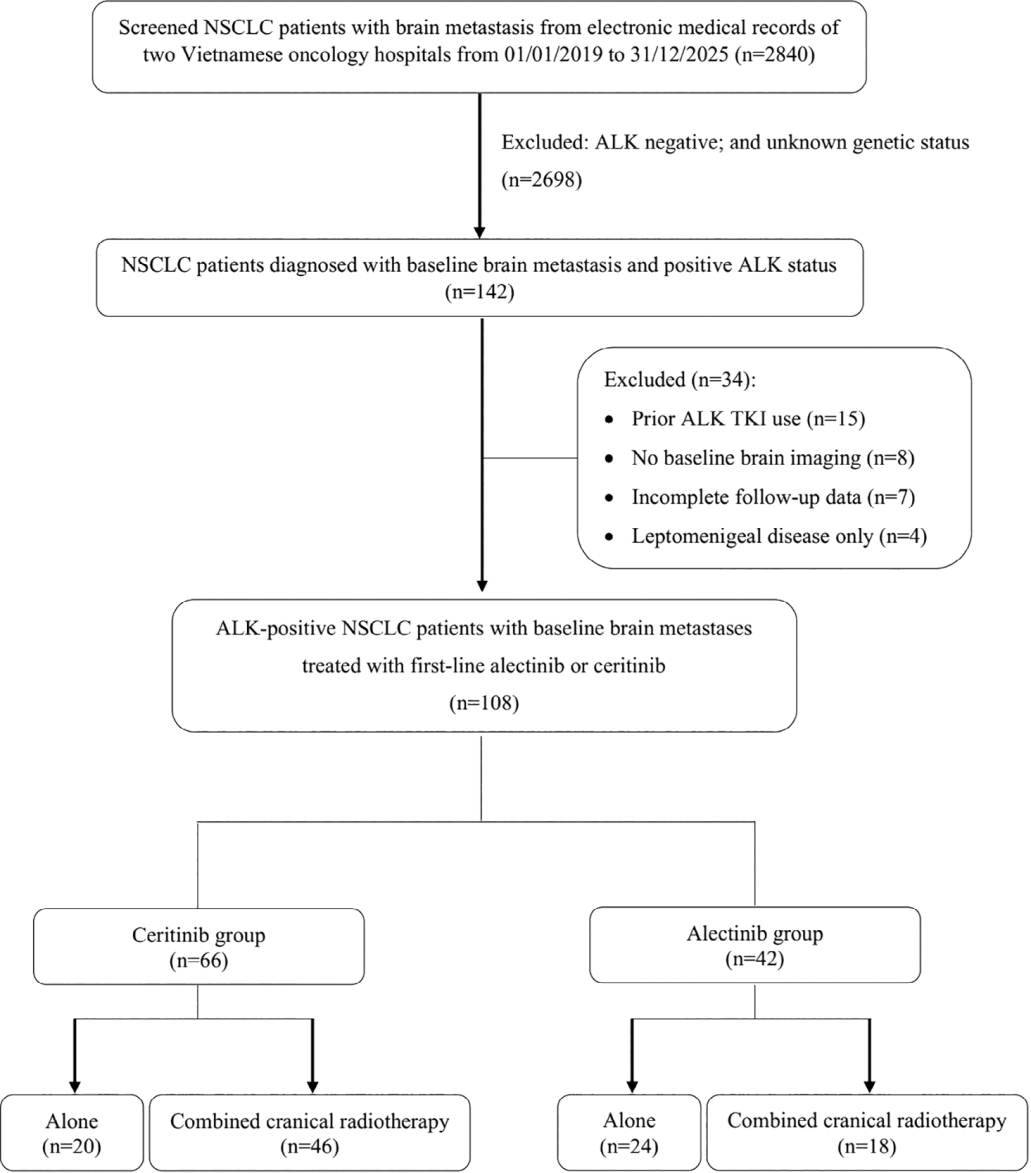
Patient flow diagram of the multicenter retrospective cohort study.

Patients were eligible if they met the following criteria: (1) histologically or cytologically confirmed advanced NSCLC; (2) brain metastases confirmed by cranial magnetic resonance imaging (MRI); (3) ALK positivity confirmed using validated methods, including fluorescence in situ hybridization (FISH), immunohistochemistry (IHC) with Ventana ALK (D5F3) antibody, real‐time reverse transcription polymerase chain reaction (RT‐PCR), or next‐generation sequencing (NGS), according to local institutional protocols at each participating center; (4) age ≥ 18 years; (5) received alectinib or ceritinib as first‐line systemic therapy for recurrent/metastatic disease; and (6) had complete clinical and imaging follow‐up records available. A total of 108 patients with ALK‐positive NSCLC and baseline brain metastases were included in the final analysis. The patient selection process is shown in Figure 1.

### 2.2 Treatment Protocol

Patients received alectinib 600 mg orally twice daily or ceritinib 450 mg orally once daily with food. Dose reduction or interruption was also performed according to the physician's judgment. Management of brain metastases was decided by a multidisciplinary tumor board comprising medical oncologists, neurosurgeons, radiation oncologists, and radiologists. Treatment options included TKI monotherapy or TKI combined with locoregional therapy (Gamma Knife radiosurgery [GKRS], whole‐brain radiation therapy [WBRT], or surgery). No patients underwent surgical resection of brain metastases. Therefore, we categorized patients into four subgroups: ceritinib alone (*n* = 20), ceritinib plus brain RT (*n* = 46); alectinib alone (*n* = 24), and alectinib plus brain RT (*n* = 18).

Data collection: All patient details were de‐identified. Baseline characteristics collected included age, sex, smoking status, Eastern Cooperative Oncology Group (ECOG) performance status, number and size of brain metastases, and presence of neurologic symptoms. We also recorded the dates of initial cancer diagnosis, diagnosis of brain metastases, initiation and discontinuation of TKI treatment, locoregional brain treatment modalities, date of systemic and CNS progression, last follow‐up, and death (if applicable). Systemic response was evaluated using contrast‐enhanced computed tomography (CT) of the chest, abdomen, and pelvis every 2–3 months using RECIST version 1.1 criteria. Intracranial response was assessed by contrast‐enhanced MRI every 6 weeks to 3 months according to the Response Assessment in Neuro‐Oncology Brain Metastases (RANO‐BM) criteria. Progression‐free survival (PFS) was defined as the interval from TKI initiation to systemic progression or death, whichever occurred first. Intracranial progression‐free survival (iPFS) referred to the time from TKI initiation to documented intracranial progression or death from any cause. Adverse events (AEs) were graded according to the National Cancer Institute Common Terminology Criteria for Adverse Events (CTCAE) version 5.0. Radiation‐related leukoencephalopathy was assessed using the Fazekas scale.

Ethical Consideration: The study was approved by the Institutional Ethics Review Board of Hanoi Medical University (decision No. 1516/GCN‐HMUIRB, dated April 26, 2024). Permission to collect and use de‐identified patient data from Vietnam National Cancer Hospital and Hanoi Oncology Hospital was granted in accordance with local regulations and institutional policies. All procedures were performed in accordance with the Declaration of Helsinki. Because of the retrospective design and the use of anonymized data, the requirement for informed consent was waived.

### 2.3 Statistical Analysis

Descriptive statistics summarized patient characteristics, with continuous variables reported as medians (interquartile range) and categorical variables as frequencies and percentages. Group comparisons utilized the *χ* ^2^ test or Fisher's exact test, as appropriate. Survival outcomes (iPFS and systemic PFS) were estimated via the Kaplan–Meier method and compared using the log‐rank test. Median survival times were reported even when they exceeded the median follow‐up duration, which is statistically valid in the presence of heavy censoring in the alectinib group. Univariable and multivariable Cox proportional hazards regression models were used to identify independent predictors of iPFS and PFS. Prespecified covariates for the multivariable models, chosen a priori based on clinical relevance and prior literature, included TKI type (alectinib vs. ceritinib), upfront brain radiotherapy (yes vs. no), the TKI × brain RT interaction term, number of brain metastases (≥ 4 vs. 1–3), ECOG performance status (≥ 2 vs. 0–1), and multi‐organ involvement (yes vs. no). These covariates were forced into the models regardless of univariable significance to permit direct comparison between endpoints and proper evaluation of effect modification. Additional variables (age, sex, smoking status, and symptomatic brain metastases) were examined in univariable analyses only. Hazard ratios (HRs) with 95% confidence intervals (CIs) were reported. No backward stepwise selection was performed. All statistical analyses were performed using SPSS software (Version 27.0; IBM Corp.) and GraphPad Prism version 10.05 (GraphPad Software). A two‐sided *p* < 0.05 was considered statistically significant.

## 3 Results

*Patient characteristics*. A total of 2840 patients with advanced NSCLC and baseline brain metastases were screened from electronic medical records of two Vietnamese oncology hospitals between January 2019 and December 2025. After excluding patients with ALK‐negative or unknown ALK status (*n* = 2698), second‐line TKI therapy (*n* = 15), absence of baseline brain imaging (*n* = 8), incomplete medical records or missing data (*n* = 7), and leptomeningeal disease only (*n* = 4), 108 patients with ALK‐positive NSCLC who received first‐line alectinib (*n* = 42) or ceritinib (*n* = 66) were included in the final analysis. The patient selection process is shown in Figure 1.

The baseline characteristics are summarized in Table 1. The median age was 55.5 years (IQR 43–63), 51.9% of patients were female, and 61.1% were never/light smokers. Most patients had good performance status (ECOG 0–1 in 86.1%) and adenocarcinoma histology (96.3%). Regarding brain metastases, 66.7% had 1–3 lesions and 33.3% had ≥ 4 lesions; symptomatic brain metastases were present in 50.9% of cases. Extracranial disease burden was considerable, with 76.9% of patients having metastases in two or more organs and 34.3% having liver involvement.

**TABLE 1:** Baseline characteristics of the 108 patients with ALK‐positive.

Characteristic	Total (*n* = 108)	Ceritinib (*n* = 66)	Alectinib (*n* = 42)	*p*
Age, years				0.174
Mean ± SD	54.0 ± 12.9	52.7 ± 11.8	56.1 ± 14.4	
Median (IQR)	55.5 (43–63)	54.0 (47–64)	57.5 (50–66)	
Sex, *n* (%)				0.844
Male	52 (48.1)	31 (47.0)	21 (50.0)	
Female	56 (51.9)	35 (53.0)	21 (50.0)	
ECOG performance status, *n* (%)				0.573
0–1	93 (86.1)	58 (87.9)	35 (83.3)	
≥ 2	15 (13.9)	8 (12.1)	7 (16.7)	
Smoking history, *n* (%)				0.841
Never or light smoker	66 (61.1)	41 (62.1)	25 (59.5)	
Ever smoker	42 (38.9)	25 (37.9)	17 (40.5)	
Histology, *n* (%)				1.000
Adenocarcinoma	104 (96.3)	63 (95.5)	41 (97.6)	
Others	4 (3.7)	3 (4.5)	1 (2.4)	
Number of brain metastases, *n* (%)				0.209
1–3	72 (66.7)	41 (62.1)	31 (73.8)	
≥ 4	36 (33.3)	25 (37.9)	11 (26.2)	
Largest brain lesion size, *n* (%)				0.519
< 1 cm	29 (26.9)	16 (24.2)	13 (31.0)	
1–3 cm	60 (55.6)	38 (57.6)	22 (52.4)	
> 3 cm	19 (17.6)	12 (18.2)	7 (16.7)	
Symptomatic brain metastases, *n* (%)	55 (50.9)	35 (53.0)	20 (47.6)	0.583
Combined brain RT, *n* (%)	64 (59.3)	46 (69.7)	18 (42.9)	0.006
Type of brain RT (among those with RT)				0.786
WBRT	23 (35.9)	17 (37.0)	6 (33.3)	
GKRS	41 (64.1)	29 (63.0)	12 (66.7)	
Number of extracranial metastatic organs, *n* (%)				0.735
0–1	25 (23.1)	16 (24.2)	9 (21.4)	
≥ 2	83 (76.9)	50 (75.8)	33 (78.6)	
Liver metastases, *n* (%)	37 (34.3)	22 (33.3)	15 (35.7)	0.811
ALK detection method, *n* (%)				0.387
NGS	80 (74.1)	50 (75.8)	30 (71.4)	
RT‐PCR	11 (10.2)	8 (12.1)	3 (7.1)	
IHC	13 (12.0)	7 (10.6)	6 (14.3)	
FISH	4 (3.7)	1 (1.5)	3 (7.1)	

Abbreviations: ALK, Anaplastic Lymphoma Kinase; ECOG, Eastern Cooperative Oncology Group; FISH, Fluorescence In Situ Hybridization; GKRS, Gamma Knife Radiosurgery; IHC, Immunohistochemistry; NGS, Next‐Generation Sequencing; RT, Radiation therapy; RT‐PCR, Reverse Transcription Polymerase Chain Reaction; WBRT, Whole Brain Radiation Therapy.

No significant differences were observed between the alectinib and ceritinib groups with regard to age, sex, smoking status, ECOG performance status, intracranial disease burden, or ALK detection method. However, combined brain RT was used significantly more frequently in the ceritinib group than in the alectinib group (69.7% vs. 42.9%; *p* = 0.006).

### 3.1 Treatment Outcomes

Treatment responses are presented in Table 2. The systemic ORR was 77.8% overall, with no significant difference observed between alectinib and ceritinib (83.3% vs. 74.2%; *p* = 0.264). Systemic DCR was also comparable (92.9% vs. 86.4%; *p* = 0.294).

**TABLE 2:** Treatment response according to RECIST 1.1 (systemic) and RANO‐BM criteria (intracranial).

Response	Total (*n* = 108)	Ceritinib (*n* = 66)	Alectinib (*n* = 42)	*p*
Systemic response, *n* (%)				
Complete response	4 (3.7)	1 (1.5)	3 (7.1)	0.158
Partial response	80 (74.1)	48 (72.7)	32 (76.2)	0.691
Stable disease	12 (11.1)	8 (12.1)	4 (9.5)	0.763
Progressive disease	12 (11.1)	9 (13.6)	3 (7.1)	0.366
Objective response rate	84 (77.8)	49 (74.2)	35 (83.3)	0.264
Disease control rate	96 (88.9)	57 (86.4)	39 (92.9)	0.294
Intracranial response, *n* (%)				
Complete response	26 (24.1)	12 (18.2)	14 (33.3)	0.076
Partial response	55 (50.9)	34 (51.5)	21 (50.0)	0.878
Stable disease	15 (13.8)	9 (13.6)	6 (14.3)	0.925
Progressive disease	12 (11.1)	11 (16.7)	1 (2.4)	0.023
Intracranial ORR	81 (75.0)	46 (69.7)	35 (83.3)	0.117
Intracranial DCR	96 (88.9)	55 (83.3)	41 (97.6)	0.026

Abbreviations: DCR, disease control rate; iDCR, intracranial disease control rate; iORR, intracranial objective response rate; ORR, objective response rate; RANO‐BM, Response Assessment in Neuro‐Oncology Brain Metastases; RECIST 1.1, Response Evaluation Criteria in Solid Tumors version 1.1.

According to RANO‐BM criteria, alectinib demonstrated a numerically higher but non‐significant iORR compared to ceritinib (83.3% vs. 69.7%; *p* = 0.117). Notably, the iDCR was significantly greater in the alectinib cohort (97.6% vs. 83.3%; *p* = 0.026). The addition of upfront brain RT did not yield a statistically significant iORR benefit in either the ceritinib cohort (71.7% with RT vs. 65.0% alone; *p* = 0.570) or the alectinib cohort (88.9% with RT vs. 79.2% alone; *p* = 0.655) (Table 3).

**TABLE 3:** Intracranial and systemic outcomes according to treatment subgroups.

Outcome measure	Ceritinib group		Alectinib group	
	Ceritinib alone (*n* = 20)	Ceritinib + RT (*n* = 46)	Alectinib alone (*n* = 24)	Alectinib + RT (*n* = 18)
iORR (%)	65.0	71.7	79.2	88.9
*p*‐value^a^	0.570		0.655	
Intracranial progression‐free survival				
Median iPFS, months (95% CI)	11.3 (5.9–16.7)	20.2 (15.5–24.9)	40.4 (30.9–49.0)	NR
Events, *n* (%)	16 (80.0)	30 (65.2)	11 (45.8)	5 (27.7)
*p*‐value^a^	0.045		0.359	
Systemic progression‐free survival				
Median PFS, months (95% CI)	8.3 (6.3–10.4)	16.6 (6.2–26.9)	28.7 (10.5–46.9)	36.7 (34.9–38.6)
Events, *n* (%)	16 (80.0)	41 (89.1)	13 (54.2)	5 (27.7)
*p*‐value^a^	0.018		0.073	

Abbreviations: iORR, intracranial objective response rate; iPFS, intracranial progression‐free survival; NR, not reached; PFS, progression‐free survival; RT, radiotherapy.

^a^ *p*‐value compared with the reference group within the same TKI. Subgroup comparisons are exploratory; interaction between TKI and brain RT was nonsignificant.

The median follow‐up duration was 36.5 months (range 5.6–54.0 months). At data cutoff, 56 progression events and only 18 deaths had occurred. Overall survival (OS) data remain immature.

**FIGURE 2:**
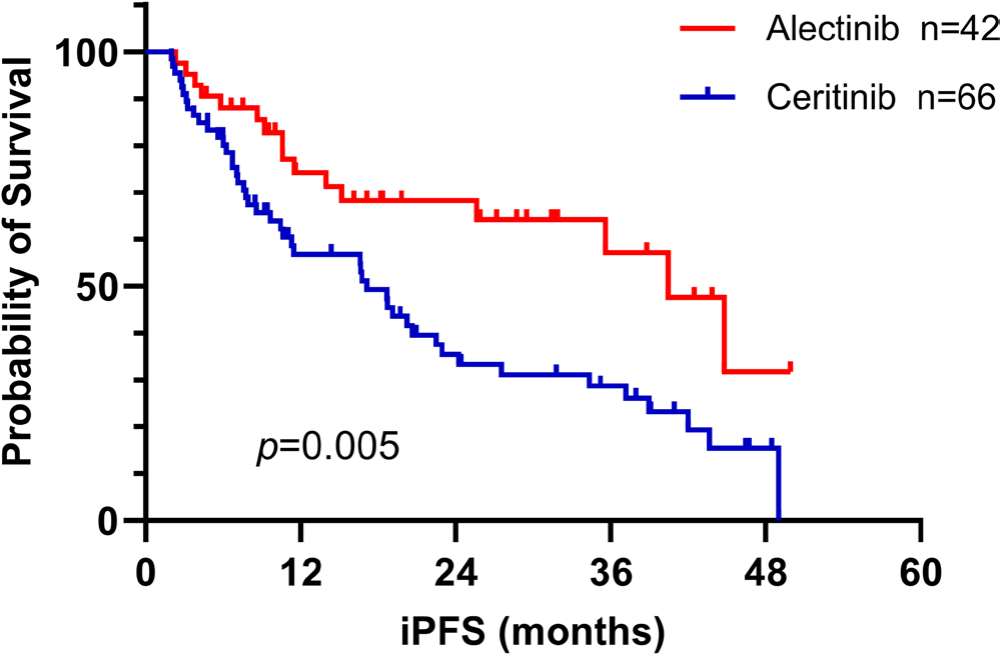
Kaplan–Meier curves of intracranial progression‐free survival (iPFS) according to first‐line TKI (alectinib vs. ceritinib).

**FIGURE 3:**
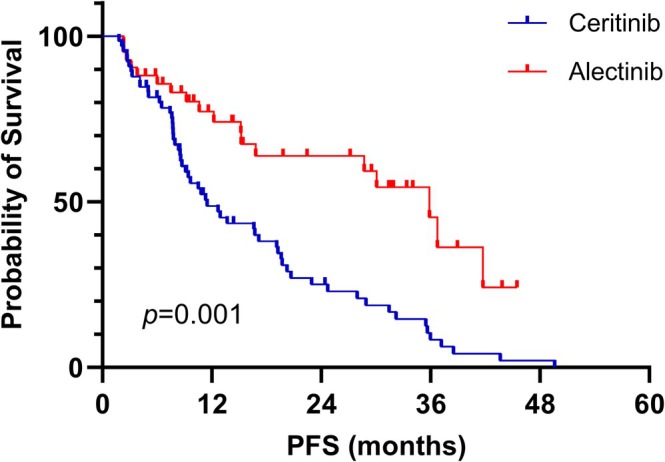
Kaplan–Meier curves of systemic progression‐free survival according to first‐line TKI (alectinib vs. ceritinib).

Alectinib was associated with significantly longer iPFS compared with ceritinib (40.4 vs. 17.2 months; HR 0.46, 95% CI 0.26–0.82; *p* = 0.005). Notably, the median iPFS in the alectinib group exceeded the overall median follow‐up duration of 36.5 months due to substantial censoring (61.9% of patients in the alectinib arm remained intracranial progression‐free at data cutoff). Systemic PFS was also significantly longer with alectinib (35.9 vs. 11.5 months; HR 0.38, 95% CI 0.22–0.65; *p* = 0.001) (Figures 2 and 3).

**FIGURE 4:**
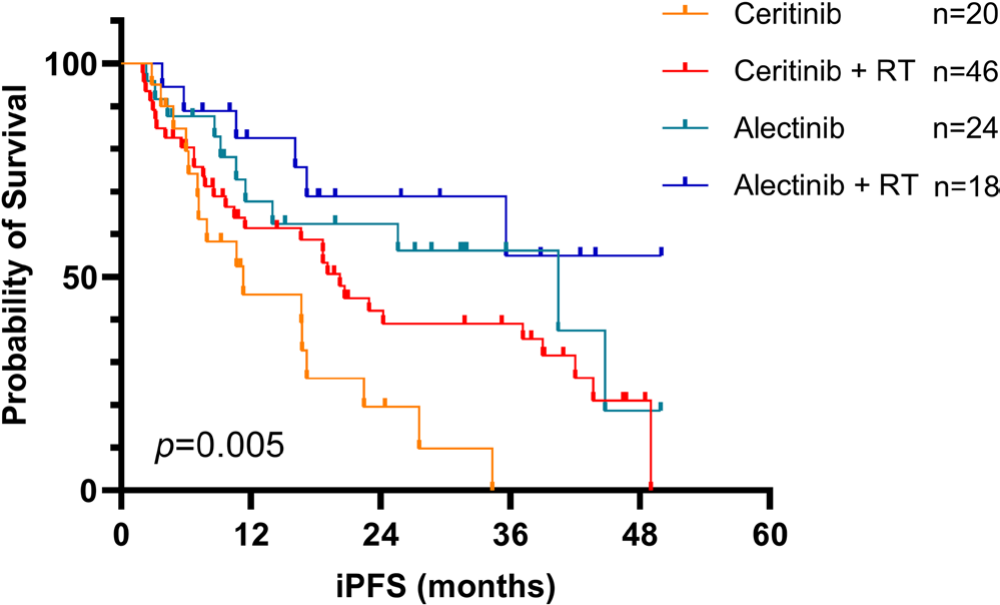
Kaplan–Meier curves of intracranial progression‐free survival (iPFS) stratified by first‐line TKI and brain RT status.

Interaction analyses indicated that the effect of upfront brain RT on iPFS and systemic PFS did not significantly differ by TKI type (p for interaction = 0.642 and 0.837, respectively). Subgroup Kaplan–Meier curves (Figure 4) and detailed outcomes (Table 3) demonstrated differing clinical trends according to upfront brain RT use within each TKI cohort. In the ceritinib cohort (*n* = 66), the addition of upfront brain RT (*n* = 46) significantly prolonged both median iPFS (20.2 vs. 11.3 months; *p* = 0.045) and systemic PFS (16.6 vs. 8.3 months; *p* = 0.018) compared with monotherapy (*n* = 20). Conversely, in the alectinib cohort (*n* = 42), upfront brain RT (*n* = 18) did not significantly improve median iPFS (NR vs. 40.4 months; *p* = 0.359) or systemic PFS (36.7 vs. 28.7 months; *p* = 0.073) relative to alectinib alone (*n* = 24).

In multivariable Cox regression analysis, first‐line alectinib remained an independent predictor of superior iPFS (HR 0.38, 95% CI 0.20–0.70; *p* = 0.002) and systemic PFS (HR 0.34, 95% CI 0.20–0.59; *p* = 0.001) after adjustment for other covariates (Tables 4 and 5). Brain RT was also independently associated with improved iPFS (HR 0.49; 95% CI 0.27–0.90; *p* = 0.023). Multi‐organ metastatic involvement was identified as an independent risk factor for inferior systemic PFS (HR 2.06; 95% CI 1.15–3.67; *p* = 0.015).

**TABLE 4:** Univariable and multivariable analysis for intracranial progression‐free survival.

Variable	Univariable HR (95% CI)	Univariable *p*‐value	Multivariable HR (95% CI)	Multivariable *p*‐value
TKI (Alectinib vs. Ceritinib)	0.46 (0.26–0.82)	0.005	0.38 (0.20–0.70)	0.002
Brain RT (yes vs. no)	0.66 (0.39–1.11)	0.118	0.49 (0.27–0.90)	0.023
TKI × Brain RT interaction	0.85 (0.32–2.25)	0.642	0.91 (0.34–2.45)	0.855
Number of brain metastases ≥ 4 (vs. 1–3)	1.21 (0.72–2.00)	0.483	1.15 (0.65–2.03)	0.628
ECOG ≥ 2 (vs. 0–1)	1.06 (0.48–2.33)	0.886	0.82 (0.35–1.92)	0.652
Multiorgan involvement (yes vs. no)	1.65 (0.88–3.06)	0.116	1.48 (0.77–2.84)	0.239
Age < 55 years (vs. ≥ 55)	0.85 (0.51–1.41)	0.528	—	—
Sex (female vs. male)	1.21 (0.72–2.01)	0.877	—	—
Smoking	1.16 (0.69–1.96)	0.58	—	—
Symptomatic brain metastases (yes vs. no)	1.13 (0.76–1.88)	0.630	—	—

Abbreviations: CI, confidence interval; ECOG, Eastern Cooperative Oncology Group; HR, hazard ratio; RT, radiotherapy; TKI, tyrosine kinase inhibitors.

**TABLE 5:** Univariable and multivariable analysis for systemic progression‐free survival.

Variable	Univariable HR (95% CI)	Univariable *p*‐value	Multivariable HR (95% CI)	Multivariable *p*‐value
TKI (Alectinib vs. Ceritinib)	0.38 (0.22–0.65)	0.001	0.34 (0.20–0.59)	0.001
Brain RT (yes vs. no)	0.76 (0.48–1.22)	0.263	0.68 (0.42–1.11)	0.124
TKI × Brain RT interaction	0.92 (0.38–2.21)	0.837	0.96 (0.39–2.35)	0.927
Number of brain metastases ≥ 4 (vs. 1–3)	1.19 (0.74–1.91)	0.473	1.10 (0.67–1.82)	0.701
ECOG ≥ 2 (vs. 0–1)	0.96 (0.48–1.93)	0.903	0.79 (0.37–1.68)	0.538
Multiorgan involvement (yes vs. no)	1.77 (1.08–3.10)	0.047	2.06 (1.15–3.67)	0.015
Age ≥ 55 years (vs. < 55)	1.14 (0.72–1.80)	0.581	—	—
Sex (female vs. male)	0.96 (0.61–1.52)	0.877	—	—
Smoking	1.06 (0.66–1.69)	0.818	—	—
Symptomatic brain metastases (yes vs. no)	1.20 (0.76–1.89)	0.448	—	—

Abbreviations: CI, confidence interval; ECOG, Eastern Cooperative Oncology Group; HR, hazard ratio; RT, radiotherapy; TKI, tyrosine kinase inhibitors.

Treatment‐Related Toxicities: AEs profiles are summarized in Table 6. Grade ≥ 3 AEs occurred in 19.0% of patients receiving alectinib compared with 34.8% on ceritinib (*p* = 0.068). Diarrhea was significantly more frequent with ceritinib (47.0% vs. 26.2%; *p* = 0.027), whereas myalgia/arthralgia was more common with alectinib (26.2% vs. 10.6%; *p* = 0.031). Rates of dose reduction (11.9% vs. 21.2%) and treatment discontinuation due to adverse events (4.8% vs. 7.6%) were numerically lower with alectinib, although the differences were not statistically significant. No treatment‐related deaths were observed in either group.

**TABLE 6:** Adverse events during treatment with alectinib or ceritinib.

Adverse event (any grade)	Total (*n* = 108)	Ceritinib (*n* = 66)	Alectinib (*n* = 42)	*p*
Any grade ≥ 3 AE, *n* (%)	31 (28.7)	23 (34.8)	8 (19.0)	0.068
Diarrhea	42 (38.9)	31 (47.0)	11 (26.2)	0.027
Nausea/vomiting	35 (32.4)	24 (36.4)	11 (26.2)	0.256
Increased ALT/AST	28 (25.9)	21 (31.8)	7 (16.7)	0.074
Fatigue	24 (22.2)	13 (19.7)	11 (26.2)	0.412
Myalgia/arthralgia	18 (16.7)	7 (10.6)	11 (26.2)	0.031
Anemia	15 (13.9)	8 (12.1)	7 (16.7)	0.492
Rash	12 (11.1)	6 (9.1)	6 (14.3)	0.378
Dose reduction due to AE, *n* (%)	19 (17.6)	14 (21.2)	5 (11.9)	0.211
Treatment discontinuation due to AE	7 (6.5)	5 (7.6)	2 (4.8)	0.547

*Note:* Data are presented as *n* (%). Adverse events (AEs) were graded according to Common Terminology Criteria for Adverse Events (CTCAE) version 5.0. One patient may have experienced multiple adverse events.

## 4 Discussion

The present study provides the first multicenter real‐world evidence from Vietnam comparing first‐line alectinib versus ceritinib in patients with ALK‐positive NSCLC and baseline brain metastases. The key findings demonstrate that alectinib was associated with significantly superior intracranial disease control (iDCR 97.6% vs. 83.3%, *p* = 0.026; median iPFS 40.4 vs. 17.2 months, *p* = 0.005) as well as systemic PFS (35.9 vs. 11.5 months, *p* = 0.001) compared with ceritinib. These benefits remained robust in multivariable analysis, independent of other prognostic factors. Alectinib also showed a numerically better tolerability profile, with fewer grade ≥ 3 AEs and lower rates of treatment discontinuation. Although the addition of upfront brain RT was associated with longer iPFS in patients receiving ceritinib, no significant benefit was observed in the alectinib group. However, formal tests for interaction between TKI type and brain RT were not statistically significant (*p* = 0.642 for iPFS and *p* = 0.837 for systemic PFS). Therefore, these findings should be interpreted with caution and considered hypothesis‐generating.

The better efficacy of alectinib observed in our study aligns with its well‐known pharmacokinetic advantages, particularly its higher unbound drug concentrations in the cerebrospinal fluid and stronger activity against ALK fusion proteins compared with ceritinib. Our results extend results from pivotal phase III trials. In the ALEX trial, alectinib demonstrated significantly better PFS (25.4 months vs. 7.4 months), and iORR (81% vs. 50%) compared with crizotinib in untreated ALK‐positive NSCLC [2]. Our real‐world alectinib cohort achieved comparable iDCR, validating the generalizability of trial findings to Vietnamese patients. Although no direct head‐to‐head phase III trial comparing alectinib and ceritinib has been conducted, the ASCEND‐4 trial showed that iORR (72.5%) and iPFS (10.7 months) were less impressive than those reported with alectinib in the ALEX trial [15]. Real‐world data from East Asia further support these observations. Multiple cohorts from Japan [16], Korea [17, 18], and China [19] have reported high iORR (range 63.6%–94.6%) and longer iPFS with alectinib in patients with baseline brain metastases. In contrast, real‐world studies of ceritinib in similar Asian populations have consistently shown lower iORR (50%–70%) and shorter iPFS [20, 21, 22]. This is likely because our patient population shares similar demographic characteristics with those in the previous studies, and all patients received first‐line treatment. Our data add meaningful real‐world evidence from an under‐represented Southeast Asian population where access to these drugs remains a major barrier.

The differential effect of adding upfront brain RT depending on the TKI used is one of the most clinically relevant findings of this study. Within the ceritinib group, the addition of brain RT appeared to improve both iPFS and systemic PFS. This result is similar to the outcomes observed when combining radiotherapy with first‐ or second‐generation EGFR tyrosine kinase inhibitors [23] In contrast, within the alectinib group, the addition of brain RT provided only marginal and non‐significant benefit. This finding is consistent with previous reports by Thomas et al. [24] and Pike et al. [25]. Combining radiotherapy with highly CNS‐penetrant targeted therapies does not appear to provide additional benefit, not only in EGFR‐mutant NSCLC but also in ALK‐rearranged disease.

Our findings provide some insights into treatment choices in resource‐limited settings. Alectinib is often more expensive and less widely available than ceritinib in many middle‐income countries, creating barriers to optimal care. In Vietnam, alectinib costs approximately four times more than ceritinib, and national reimbursement policies tend to favor the less expensive option. Our data suggest that ceritinib combined with upfront brain radiotherapy may be a reasonable alternative when alectinib is inaccessible due to cost or availability constraints. Notably, upfront brain radiotherapy was used substantially more frequently in the ceritinib group than in the alectinib group (69.7% vs. 42.9%), reflecting real‐world physician preference in this cohort. Formal cost‐effectiveness analysis was beyond the scope of this study. In contrast, alectinib achieved a high rate of intracranial disease control, which may allow brain radiotherapy to be safely deferred until disease progression, potentially sparing patients from long‐term neurocognitive side effects. However, the optimal timing of upfront brain radiotherapy before initiating alectinib remains controversial. Due to the small sample size in the alectinib plus brain RT subgroup (*n* = 18), this analysis had limited statistical power to detect small or moderate differences; therefore, the absence of additional benefit with radiotherapy in the alectinib group should be interpreted with caution. Additional data from larger prospective studies, such as the ongoing DURABLE trial, are needed to further clarify the role of upfront brain RT in patients treated with alectinib [26].

This study has several limitations that warrant cautious interpretation of the findings. First, its retrospective nature inherently introduces selection bias and confounding by indication. Both the choice of first‐line TKI (alectinib or ceritinib) and the decision to administer upfront brain RT were non‐randomized. These decisions were likely influenced by physician preference, drug availability, reimbursement policies, and evolving perceptions of CNS efficacy during the study period. This is reflected in the significantly higher use of brain RT in the ceritinib group. Although we performed multivariable adjustment and formal interaction testing, residual confounding may remain. Second, the relatively small sample size—particularly within the alectinib plus upfront brain RT subgroup (*n* = 18)—limits the statistical power of certain subgroup comparisons and interaction analyses. Third, ALK rearrangement was confirmed using multiple diagnostic assays (next‐generation sequencing, RT‐PCR, immunohistochemistry with Ventana D5F3, and FISH) according to local institutional protocols at the two participating centers. Although the distribution of testing methods was balanced between the alectinib and ceritinib groups (*p* = 0.387), inter‐assay variability in sensitivity and specificity may have influenced patient selection and represents a potential source of bias. Fourth, intracranial and systemic response assessments were performed locally without central radiological review. Although contrast‐enhanced brain MRI was standardly utilized at both centers, imaging intervals were not strictly uniform. Furthermore, reliance on routine clinical records for data extraction may have introduced documentation inconsistencies and potential under‐reporting of low‐grade adverse events. Fifth, patients were enrolled from only two tertiary oncology centers in northern Vietnam, which may limit the generalizability of the findings to other regions or healthcare settings. Finally, overall survival (OS) data remain immature due to the low number of mortality events (*n* = 18) and the relatively short median follow‐up duration of 36.5 months. Notably, the median iPFS in the alectinib arm (40.4 months) exceeded the overall median follow‐up time. While this is a recognized phenomenon in survival analyses when a large proportion of patients remain progression‐free at data cutoff—and is appropriately accounted for by the Kaplan–Meier estimator—it warrants cautious interpretation. This immaturity of OS data is further compounded by the potential crossover of patients to third‐generation ALK TKIs upon disease progression.

In this real‐world multicenter cohort of Vietnamese patients with ALK‐positive NSCLC and baseline brain metastases, first‐line alectinib demonstrated significantly better intracranial and systemic efficacy compared with ceritinib. Although upfront brain radiotherapy showed a potential trend toward improving outcomes in the ceritinib group, the lack of a significant interaction between TKI type and radiotherapy precludes definitive conclusions regarding a differential benefit. Therefore, while alectinib should be the preferred first‐line option when accessible, the combination of ceritinib and upfront brain radiotherapy remains a pragmatic and reasonable alternative in resource‐constrained settings. Larger prospective studies are warranted to validate these hypothesis‐generating findings.

## Author Contributions

**Hoang Gia Nguyen:** conceptualization, methodology, data curation, writing – original draft, writing – review and editing, investigation. **Xuan Hau Nguyen:** conceptualization, methodology, writing – review and editing, supervision, investigation. **Thu Huong Nguyen:** data curation, formal analysis, validation, writing – review and editing. **Quang Bui Vinh:** conceptualization, investigation, writing – review and editing, methodology, supervision, data curation. **Phuong Nguyen:** data curation, validation, formal analysis, writing – review and editing, software.

## Funding

The authors have nothing to report.

## Ethics Statement

The study was approved by the Institutional Ethics Review Board of Hanoi Medical University (Approval No. 1516/GCN‐HMUIRB, dated April 26, 2024). Permission to collect and use de‐identified patient data from Vietnam National Cancer Hospital and Hanoi Oncology Hospital was granted in accordance with local regulations and institutional policies. All procedures were performed in accordance with the Declaration of Helsinki. Because of the retrospective design and the use of anonymized data, the requirement for informed consent was waived.

## Conflicts of Interest

The authors declare no conflicts of interest.

## Data Availability

The data that support the findings of this study are available from the corresponding author upon reasonable request.
